# TiS_3_ Nanoribbons: A Novel Material for Ultra-Sensitive Photodetection across Extreme Temperature Ranges

**DOI:** 10.3390/s23104948

**Published:** 2023-05-21

**Authors:** Mohammad Talib, Nishant Tripathi, Samrah Manzoor, Prachi Sharma, Vladimir Pavelyev, Valentyn S. Volkov, Aleksey V. Arsenin, Sergey M. Novikov, Prabhash Mishra

**Affiliations:** 1 Centre for Nanoscience and Nanotechnology, Jamia Millia Islamia (A Central University), New Delhi 110025, India; 2 Samara National Research University, 34, Moskovskoye Shosse, Samara 443086, Russia; 3 School of Electronics Engineering (SENSE), Vellore Institute of Technology (VIT), Vellore 632014, India; 4 IPSI RAS—Branch of the FSRC “Crystallography and Photonics” RAS, Molodogvardeyskaya 151, Samara 443001, Russia; 5 Center for Photonics & 2D Materials, Moscow Institute of Physics and Technology (MIPT), Dolgoprudny 141700, Russia; volkov.vs@mipt.ru (V.S.V.);; 6 Laboratory of Advanced Functional Materials, Yerevan State University, Yerevan 0025, Armenia

**Keywords:** TiS_3_ nanoribbons, wide operating temperature photodetector, cryogenic temperature, elevated temperature

## Abstract

Photodetectors that can operate over a wide range of temperatures, from cryogenic to elevated temperatures, are crucial for a variety of modern scientific fields, including aerospace, high-energy science, and astro-particle science. In this study, we investigate the temperature-dependent photodetection properties of titanium trisulfide (TiS_3_)- in order to develop high-performance photodetectors that can operate across a wide range of temperatures (77 K–543 K). We fabricate a solid-state photodetector using the dielectrophoresis technique, which demonstrates a quick response (response/recovery time ~0.093 s) and high performance over a wide range of temperatures. Specifically, the photodetector exhibits a very high photocurrent (6.95 × 10^−5^ A), photoresponsivity (1.624 × 10^8^ A/W), quantum efficiency (3.3 × 10^8^ A/W·nm), and detectivity (4.328 × 10^15^ Jones) for a 617 nm wavelength of light with a very weak intensity (~1.0 × 10^−5^ W/cm^2^). The developed photodetector also shows a very high device ON/OFF ratio (~32). Prior to fabrication, the TiS_3_ nanoribbons were synthesized using the chemical vapor technique and characterized according to their morphology, structure, stability, and electronic and optoelectronic properties; this was performed using scanning electron microscopy (SEM), transmission electron microscopy (TEM), Raman spectroscopy, X-ray diffraction (XRD), thermogravimetric analysis (TGA), and a UV–Visible–NIR spectrophotometer. We anticipate that this novel solid-state photodetector will have broad applications in modern optoelectronic devices.

## 1. Introduction

Photodetectors are very useful devices for obtaining information from the light beam. When the light beam interacts with any object, the crucial information regarding the object is stored in the reflected/transmitted light from the object. To retrieve information from the light beam, photodetectors are required [1,2]. Photodetectors can be used in various applications, such as satellite communication [1], quantum computing [3,4], magnetic recording [5], optical communications [6], imaging [1,7], medicine [8], chemistry [8], biochemistry [8], healthcare [8,9], environmental control [6], spectroscopies [2], robotics applications [10], and so on. 

In the majority of applications in which photodetectors are required, the parameters and environmental conditions are different. For example, applications such as flame detection, space exploration, night vision and combustion, and applications related to chemical reactions, gas turbines, geothermal, gas and oil exploration, etc., are related to a high climatic temperature; therefore, photodetectors involved in the mentioned applications require a high operating temperature [2,11,12,13]. Some applications, such as space exploration, arctic research, cryostats, high-energy physics, astroparticle physics, Cherenkov radiation, fluorescence sensors, remote sensing in space, etc., belong to low/cryogenic environmental temperature conditions [2,13]. Therefore, photodetectors that are operable in low/cryogenic temperatures are highly required for such types of applications. In conclusion, we can say that photodetectors that are operable in wide operating temperatures are very crucial. Many materials, such as colloidal quantum dots [14], SiCN/Si MSM [11], InGaN [11], GaN [13], InGaAs/InP [15], InAs/GaSb [15], the InAs–GaAs vertical quantum dot [16], the GaAs/AlGaAs quantum well [17], CNTs [2], etc., have been reported to be applicable in the fabrication of photodetectors for cryogenic, as well as for their elevated temperature. There are many problems that arise when attempt to develop photodetectors that have wide operating temperatures. In the case of cryogenic operations, the main limitations are the occurrence of non-radiative recombination, such as Auger recombination, increments in the bandgap of materials and a low charge carrier mobility. Due to these limitations, the value of photocurrents decreases [18,19,20]. In the case of elevated temperatures, the performance of photodetectors is degraded due charge carrier scattering, drastic increments in the dark current and the generation of defects in materials [2,21,22]. Apart from the mentioned issues, photodetection materials have other general limitations, such as a low capacity for absorbing the photons, a narrow limit for absorbing the wavelength of light, a slow response/recovery time, etc.

In order to minimize the above-mentioned limitations, and thus develop a high-performance photodetector with a wide operating temperature range, we explore the TiS_3_-nanoribbons as a light-sensing material. TiS_3_ is known as a very new member of the transition metal chalcogenide family. It has a layered structure, and individual layers are attached to each other by a weak Van der Waals force; therefore, it is easy to exfoliate TiS_3_ from its bulk part into a few-layered structure, but its individual layers are very stable in nature. Generally, it shows a bandgap energy of approximately 1.1 eV, but the bandgap energy can be changed by manipulating its structure [23]. Due to the narrow bandgap, TiS_3_ is capable of absorbing a wide range of solar spectra with high efficiency. TiS_3_ nanostructures are naturally found in n-type direct bandgap semiconducting materials. The big advantage of TiS_3_ nanostructures is that they have very high electronic mobility (~10,000 cm^2^/V·s (theoretically), 25–80 cm^2^/V·s (experimentally)) [24,25]. TiS_3_ shows very high in-plane anisotropy, as well as different values of electronic and thermal coefficients on the different axis [26]. The high anisotropy in the thermal and electronic coefficients reduces the hot electrons, and hence, the backscattering of charge carriers [26]. Generally, TiS_3_ nanostructures have very high stability against chemical and thermal effects, but at very high temperatures, some structural defects start to be created [27]. TiS_3_ has already shown its applicative potential in the fabrication of broadband photodetectors [28,29], but to the best of our knowledge, there are no reports available on their operating temperature-dependent photodetection properties. Therefore, due to its high stability, electronic conductivity, thermal and electronic anisotropy, narrow bandgap, and tunable electronic and optoelectronic properties, TiS_3_ could be a suitable material in high-performance photodetectors with wide operating temperatures. 

In the present article, a TiS_3_ nanoribbon-based photodetector with a wide operating temperature (77 K to 543 K) is developed. A detailed study of its temperature-dependent photodetection properties is conducted. A solid-state device is developed by using standard photolithography and dielectrophoresis techniques. Before developing the device, TiS_3_ nanoribbons are fabricated via the sulfurization of titanium powder by using the chemical vapor transportation technique. The TiS_3_ nanoribbons are investigated for their morphological, structural, electronic and optoelectronic properties using SEM, TEM, Raman spectroscopy, XRD, a UV–Visible–NIR spectrophotometer and TGA. Important parameters, such as its photoresponsivity, quantum efficiency and detectivity, are observed and a sensing mechanism is established with proper justification and results.

## 2. Experimental Section

The TiS_3_ nanoribbons were synthesized via the sulfurization of titanium (2 gm, 99.95% purity) with sulfur (6 gm, 99.95% purity) in a vacuum-sealed quartz ampule at a temperature of 500 °C using a 20 h heating time. The pressure inside the ampule was maintained at 2 × 10^−5^ mbar and the sulfurization process was conducted by employing the chemical vapor transportation technique. Before the fabrication of the solid-state device, the prepared TiS_3_ nanostructure was investigated for its morphological, structural, electronic and optoelectronic properties using SEM, TEM, Raman spectroscopy, XRD, a UV–Vis–NIR spectrophotometer and TGA. The solid-state device was fabricated via the deposition of Ag (100 nm)/Ti (8 nm) interdigitated electrodes (IDEs) on a thermally oxidized silicon wafer using a standard photolithography technique. Simultaneously, a dispersion of TiS_3_ nanostructures was prepared in isopropyl alcohol (IPA) by using a high-power probe ultrasonicator. Finally, the photodetector was developed via the deposition of dispersed TiS_3_ nanostructures on the IDEs deposited on the solid-state device by using the dielectrophoresis technique. A detailed description of the process of fabricating the TiS_3_ nanostructures and the devices based on them is presented in our previous article [28]. The photodetection properties of the as-developed photodetector were investigated via illumination with 617 nm wavelengths of laser light. The photodetection measurement was performed as a function of the power density of light at different operating temperatures. All chemicals used in the experiment were bought from Sigma-Aldrich (St. Louis, MO, USA).

## 3. Result and Discussion 

### 3.1. Material Characterization

**Figure 1:**
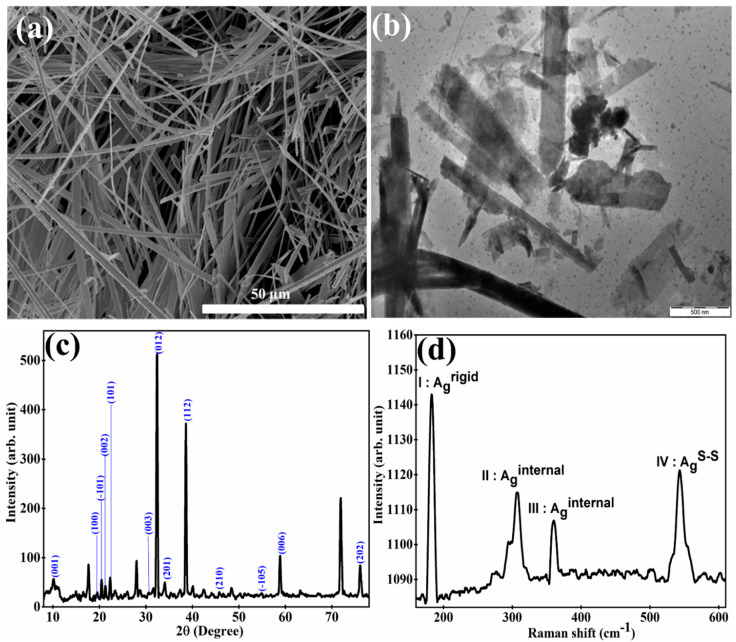
(**a**–**d**) represents the SEM image, TEM image, XRD pattern and Raman spectra for the as-synthesized TiS3 nanoribbons, respectively.

**Figure 2:**
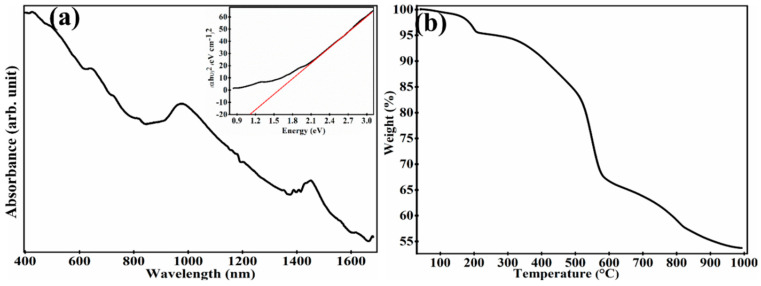
(**a**) Shows the absorbance spectra with a Tauc plot in the inset (the black line shows the plot of (αhυ)^2^ (eV cm^−1^)^2^) vs. Energy (eV) while red lone shows the tangential of the plot on x-axis), while (**b**) represents the TGA analysis for the as-prepared TiS3 nanoribbons.

**Figure 3:**
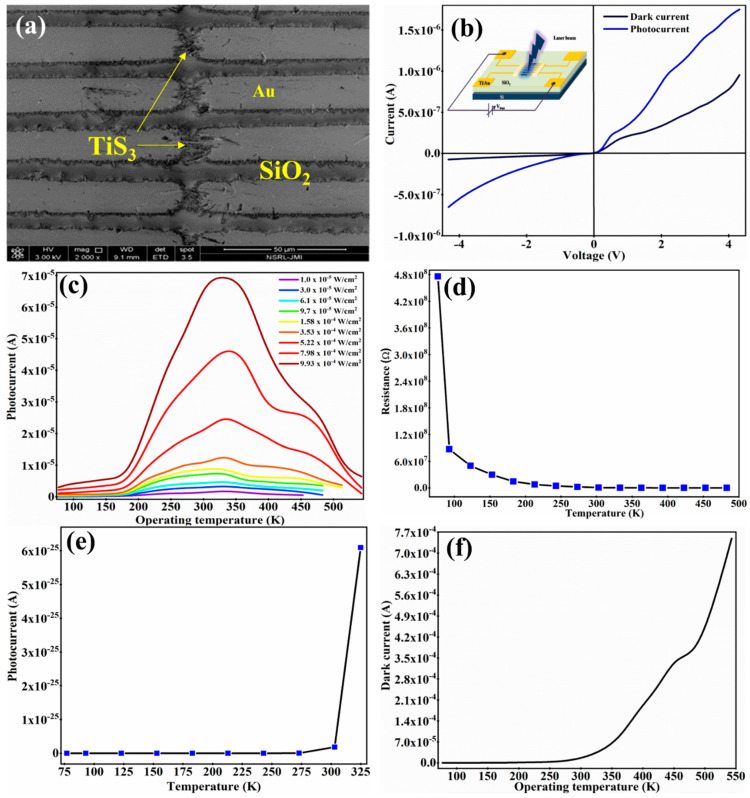
(**a**) Shows the SEM image of the prepared solid-state device. (**b**) I–V characteristics of as-developed solid-state device in dark as well under the illumination of 617 nm laser light. Inset of (**b**) represents the schematic diagram of the technique used for the analysis of the photodetection properties of TiS_3_ nanoribbons. (**c**) Represents the measurement of the photocurrent as a function of the operating temperature and power density of light. (**d**) Shows the resistance of the device as a function of the operating temperature. (**e**) Represents the graph of the photocurrent vs. operating temperature based on Equation (3). (**f**) Shows the plot for the dark current at different operating temperatures.

To understand their morphological properties, the prepared TiS_3_ nanostructures were investigated using scanning electron microscopy (SEM) (Figure 1a) and transmission electron microscopy (TEM) (Figure 1b). To perform SEM and TEM analysis, a dispersion was prepared by mixing 10 mg of TiS_3_ nanostructures in 25 mL of isopropyl alcohol. The TiS_3_ nanostructures were deposited on a silicon wafer, as well as on a copper grid, using the drop-casting process in order to investigate the sample using SEM and TEM, respectively. It is clearly observable from the SEM and TEM images that the as-fabricated TiS_3_ nanostructures have a well-uniformed nanoribbon-type shape. The prepared nanoribbons have a width in the range of 30 nm to 57 nm and a length in the range of 200 nm to 10,000 nm. The TEM image reveal that the thickness of the TiS_3_nanoribbons is in the range of 10 nm to 57 nm. One important point has been noticed: the length of the nanoribbons in the TEM image is shorter than those in the SEM image. In order to investigate the TiS_3_ nanostructures using TEM, we have to prepare the sample on a copper grid. The steps involved in the sample preparation can break the TiS_3_ nanoribbons into smaller sizes. This could be considered a possible reason for the size difference between the structures in the SEM and TEM images. In order to obtain phase and structural property information, the X-ray diffraction (XRD) pattern was obtained for the prepared TiS_3_ nanostructures with Cu Kα radiation (λ = 1.54 Å) in a θ–2θ configuration. The XRD pattern can be seen in Figure 1c. The important peaks with their crystalline phases of TiS_3_ nanostructures can be seen in Figure 1c. All peaks were found to be similar to previously reported data [30]. In order to obtain further information about the structural quality, the as-developed TiS_3_-nanoribbons were investigated via Raman spectroscopy. A sharp and intense peak is clearly visible in the Raman spectra of Figure 1d at 169 cm^−1^, 298 cm^−1^, 369 cm^−1^ and 550 cm^−1^, which correspond to the I-A_g_ ^rigid^, II-A_g_ ^internal^, III-A_g_ ^internal^ and A_g_ ^S-S^. The I-peak is a signature of the TiS_3_ nanostructure; it is a unique peak that occurs only in TiS_3_ nanostructures. The I-peak, A_g_ ^rigid^, shows the out-of-phase vibrations of the TiS_3_ chains along the b-direction. The II-peak and III-peak come into the picture due to the internal vibrations of individual layers of TiS_3_. The IV-peak in the TiS_3_ Raman spectra represents the in-plane out-of-phase vibrations of S–S molecules [23]. The difference between the position of I-peak and III-peak is used to estimate the number of layers in the TiS_3_ nanostructures [31]. All the peaks in the present work are highly matched with existing data, which verifies that the as-prepared TiS_3_ nanostructures are highly pure and have a very good structural quality [23]. To obtain the optical properties, the as-developed TiS_3_-nanoribbons were analyzed using a UV–Visible–NIR spectrophotometer. The absorbance spectra shows the strong peaks centered around 418 nm, 629 nm, 976 nm, 1450 nm and 1625 nm (Figure 2a). TiS_3_ nanostructures have the ability to absorb the light in the UV–Vis–NIR range. The bandgap energy of the as-prepared TiS_3_ nanostructures was estimated using the Tauc plot method (inset of Figure 2a). The bandgap energy was found to be around 1.11 eV. To obtain information about the thermal stability, thermogravimetric analysis (TGA) was performed on the prepared TiS_3_ nanostructures. TGA analysis was conducted by heating the TiS_3_ nanostructures from room temperature to 1000 °C while the change in weight% of the TiS_3_ nanoribbons was monitored (see Figure 2b). TGA analysis showed no significant loss in weight% of the TiS_3_ nanoribbons up to 170 °C, which means that the as-prepared TiS_3_-nanoribbons are very stable up to 170 °C. After 170 °C, the weight% started to decrease. From 170 °C to 275 °C, the weight% decreased to 94%. When the TiS_3_ nanoribbons were heated beyond 275 °C, the weight% started to decrease sharply. The reduction in weight% happened due to the removal of S atoms from the TiS_3_ nanoribbons. It has been reported that during heat treatment, TiS_3_ nanostructures start to convert into TiS_2_ and then TiO_2_ nanostructures, which could be considered a major reason for the weight% loss [32,33,34]. After analyzing their morphological, structural and optical properties in detail, the prepared TiS_3_ nanostructures were investigated for their operating temperature-dependent photodetection properties. The inset of Figure 3a shows the schematic diagram of the technique used for the photodetection analysis of an as-developed solid-state device based on the TiS_3_ nanostructures. The photodetection properties of the as-developed solid-state device was investigated as the function of the power density of light, as well as operating temperature. Figures S1 and S2 (Supplementary information (SI)) show the real-time measurements of the photocurrent in the multiple cycles in the operating temperature range of 77 K to 543 K, with the power density of light maintained in the range of 1.0 × 10^−5^ W/cm^2^ to 9.93 × 10^−4^ W/cm^2^. All photodetection measurements were observed with a 5 V bias voltage and with a 617 nm wavelength of laser light. Figure S1a–i (SI) represent the real-time photocurrent measurement as a function of the power density of light in an operating temperature range of 77 K to 303 K, with an increment interval of 30 K. Figure S2a–h (SI) show the real-time photocurrent measurement for the operating temperature range of 333 K to 543 K, with increment steps of 30K. It is clearly observable from Figures S1 and S2 (SI) that the as-developed photodetector shows a very stable and repeatable response in the operating temperature range of 77 K to 453 K. Beyond an operating temperature of 453 K, the developed photodetector shows less sensitivity to a low power density of light, but at a higher power density of light, it shows a stable and repeatable result. As we can see from Figure S2f (SI), at an operating temperature of 483 K, the as-developed photodetector became insensitive to a power density of light that was less than 3.0 × 10^−5^ W/cm^2^. In the same pattern, when the operating temperature was further increased, the light power density cut-off value was increased. In addition, one important point to be noted is that, at an operating temperature of 543 K, the base/dark current was found to be continuously increasing. In order to understand the reason behind the obtained photocurrent pattern, the developed device was further investigated. In general, the production of photocurrent occurs mainly due to the photo-conductance, photo-voltaic and photo-thermoelectric effect. Figure 3a shows the SEM image of the prepared device based on the TiS_3_ nanoribbons. To obtain a clear picture of the sensing mechanism, the current–voltage characteristics of the developed device were taken at room temperature in the dark, as well as under laser light illumination (see Figure 3b). The absence of a short circuit current in the I–V characteristics withdrew the possibility of a photo-voltaic effect [35]. Since the device is uniformly illuminated by laser light, in the case that the current is produced by the photo-thermoelectric effect, the produced current travels in the opposite direction, and due to the equal magnitude, the current is cancelled out at the TiS_3_–electrode interface [36]. Therefore, it can be considered that photocurrent generation took place due to the photoconductance effect. In addition, the non-linear behavior of the current, with respect to bias voltage, is clearly observable in the I-V characteristics graph of Figure 3b; this represents the fabrication of Schottky contacts at the TiS_3_–nanoribbon–electrodes interface [2].

### 3.2. Investigation of Photoresponse 

Photoconductance is a well-known mechanism in light-sensing devices. In the case of photoconductance, the photosensitive material absorbs the illuminated photons, which have an energy equal to or greater than the bandgap energy of the light-sensitive material. By this process, electron–hole pairs are generated in the light-sensitive material and, after obtaining sufficient energy, photoinduced electrons jump from the valence band to the conduction band. Later, the as-produced electron and hole pairs are separated by an external bias voltage and are absorbed on opposite terminals of the battery. Due to the entire process, the photocurrent starts to flow in the electronic circuit [2]. Figure 3c shows the combined graph for the photocurrent of the as-developed device as a function of the operating temperature at different power densities of light. It can be clearly observed in the figure that the values of the photocurrent increased sharply when the value of the operating temperature increased from 73 K to 333 K, and that when the value of the operating temperature increased further, the values of the photocurrent started to decrease. The same pattern was observed for all values of the power density of light. The various effects of the power density of light on the performance of the TiS_3_ nanoribbon-based photodetector were explained in detail in our previous work [28]. Here, our focus is only on the various effects of the operating temperature on the performance of photodetectors. To understand clearly, the performance of a photodetector as a function of the operating temperature can be divided into two ranges of operating temperatures; the first is 77 K to 333 K, and the second is 333 K to 543 K. First, we will understand the various effects of the operating temperature from 77 K to 543 K. As explained in the Section 1, TiS_3_ is known as a narrow-bandgap n-type semiconductor. In such types of semiconductors, when the temperature increases from 77 K to 150 K, the mobility of the charge carriers starts to increase sharply. In addition, when the temperature increases further from 150 K to 333 K, the mobility of the charge carriers starts to decrease slowly. The maximum value of mobility has been reported for a 150 K operating temperature [37]. The increment in the mobility decreases the transition time (τ_trans_) of the charge carriers. Mathematically, the situation can be understood using the following expression [2]:

(1) $$\tau_{\mathrm{trans}}=\frac{L^{2}}{µ.\;\;V_{bias}}$$

As we can see in the above expression, the value of τ_trans_ depends on the channel length (L), mobility of the charge carriers (µ) and bias voltage. The channel length (~5 µm) of the device and bias voltage (5 V) remained constant during all of the measurements. Therefore, mobility is a measure factor that can influence the τ_trans._ In addition, τ_trans_ time directly influences the photoconductive gain (G) of a photodetector. The photoconductive gain can be calculated by the following equation [2]:

(2) $$G=\frac{\tau_{life}}{\tau_{trans}}$$

where τ_life_ is the lifetime of charge carriers. Therefore, when the operating temperature of the device increased from 77 K to 150 K, the mobility of the device increased sharply, and the mobility decreased the value of τ_trans._ The decrease in the value of τ_trans_ increased the photoconductive gain of the as-prepared device and, hence, the value of the photocurrent increased. The increment in the value of the photocurrent is almost linear [2,38,39]. Beyond a 150 K operating temperature, the mobility of the TiS_3_ nanostructures starts to decrease slowly [37]. To understand the behavior of the photocurrent beyond the 150 K operating temperature, we have to consider the other factors. One very important factor involved in narrow-bandgap n-type materials is the non-radiative recombination of the charge carriers. Due to the direct Auger recombination of photo-induced charge carriers, non-radiative recombination becomes a major concern at cryogenic temperatures. This might also be a reason for the low photocurrent at a 77 K operating temperature [18]. When the device temperature increases, the non-radiative recombination starts to decrease, which enhances the value of the photocurrent with respect to temperature. Another reason might be the instability in the bandgap of narrow-bandgap materials at the cryogenic temperature. It has been reported that when the temperature of narrow-bandgap materials decreases towards the cryogenic temperature, then the bandgap energy starts to increase [19,20]. Therefore, the increment in the bandgap energy and disturbance in the band structure of TiS_3_ nanoribbons at the cryogenic temperature could be considered another reason for the low photocurrent at 77 K and the increment in the photocurrent with respect to the increment in the operating temperature. As explained earlier, the absorbance spectra of TiS_3_ nanoribbons have one strong peak centered around 629 nm. Therefore, due to disturbance in the band structure, it is possible that the photon absorbance peak and capacity shifted at a 77 K operating temperature. However, when the temperature of the device increased from 77 K to higher values, then the band structure of the TiS_3_ nanostructures started to return to its actual form, which can enhance the photocurrent values. The last important factor is the resistance of the device. It is a well-known phenomenon that the resistance of the semiconducting materials starts to increase with the decrease in the temperature. Figure 3d shows the resistance of an as-developed device with respect to the increment in temperature. As we can see, the resistance of the device decreases exponentially with respect to the increment in the operating temperature. The resistance of the device was found to be very high at a 77 K operating temperature, and sharply decreased with the increment in the temperature up to 150 K. When the device temperature increased beyond the 150 K, a slow decrease in the resistance was noticed. Therefore, beyond a 150 K operating temperature, the device starts to behave completely according to photoconductive behavior. In addition, the photocurrent starts to increase sharply with non-linear behavior after a 150 K operating temperature. All of the above-mentioned factors could be the reason for the photocurrent pattern found in the TiS_3_ nanoribbon-based photodetector; indeed, all mentioned factors played an important role in the generation of the photocurrent with respect to an operating temperature of 77 K to 333. K. Manik et al. reported a detailed analysis of the behavior of photodetectors in the operating temperature range of 77 K to 325 K [40]. They developed the following relation between the operating temperature (T), bandgap energy (E_g_) and Boltzmann constant (K) for the generation of photocurrent (i_ph_):

(3) $$i_{ph}\sim T^{-3}\exp\left(-E_{g}/KT\right)$$

Based on the above relation, we tried to plot the pattern of the photocurrent for our case. Figure 3e shows the pattern of the photocurrent based on Equation (3). It was found that the pattern of the photocurrent based on Equation (3) is well matched with the TiS_3_ nanoribbon-based photodetector, as presented in Figure 3c. The matching of the photocurrent behavior also validates our proposed physics regarding the behavior of the TiS_3_ nanoribbon-based photodetector in the operating temperature range of 77 K to 333 K. Now, the behavior of the photocurrent beyond a temperature of 333 K will be explained. As we can see in Figure 3c, the value of the photocurrent starts to decrease with respect to the increment in the operating temperature beyond the temperature of 333 K. It was observed that the photocurrent decreases slowly up to 422 K and later it starts to decrease rapidly. The mobility of the charge carriers is highly affected by the temperature. It has been reported that the mobility of the charge carriers in TiS_3_ nanoribbons starts to decrease sharply after a 333 K annealing temperature [37]. As per the above discussion, the mobility of charge carriers affects the value of τ_trans_, and hence, the value of the photoconductive increases. Therefore, when the mobility of the charge carriers starts to decrease slowly above the 150 K temperature, the value of the photocurrent starts to be affected accordingly. It has been reported that beyond 393 K, the mobility of narrow-bandgap 2D materials starts to decrease drastically with the factor of T^−2^ [41]. Therefore, this decrement in the mobility, which is at first slow and then rapid, could be considered as one reason for the pattern in the photocurrent after a 333 K operating temperature (Figure 3c). Another reason could be considered the charge carrier scattering. It has been reported that in narrow-bandgap 2D materials, at elevated temperatures the charge carrier scattering increases sharply. The scattering of charge carriers mainly took place in the form of electron-grain boundary scattering, electron–phonon scattering and electron-defects scattering. Due to the various types of scattering at elevated operating temperatures, the τ_trans_ for charge carriers increased; this decreased the photoconductance gain and hence the value of the photocurrent [35]. Another disadvantage of scattering is that it increases the dark current of the device. At higher temperatures, electron–phonon scattering increases drastically, which increases the transit time of charge carriers in the channel. Enhancing the transit time can increase the possibility of the recombination of the charge carriers and the dark current of the device [42]. Since TiS_3_ is a narrow-bandgap material, thermionic emission can also play a role in the increment of the dark current [43]. Figure S3 (SI) shows the I–V characteristics of the as-prepared photodetector in dark conditions. In addition, Figure 3f shows the graph for the measurement of the dark current as a function of the operating temperature. It is clearly observable from Figure S3 (SI) and Figure 3f that the dark current of the device increases with respect to the increment in the operating temperature. The increment in the value of the dark current was found to be very small, almost negligible, from a 77 K to 333 K operating temperature. When the operating temperature increased beyond 333 K, the value of the dark current almost increased with an exponential pattern. The drastic increment in the dark current reduces the sensitivity and hence the photocurrent of the device. It was noticed that the value of the dark current became very high after a 453 K operating temperature. Due to the high dark current, the as-developed photodetector started to be insensitive towards the low power density of light. However, at higher power densities of light, a stable photocurrent was evident. As we can see in Figure 3c, the as-developed photodetector was unable to detect light that had a power density less than 1.0 × 10^−5^ W/cm^2^ at a 483 K operating temperature; meanwhile, at a 543 K operating temperature, the as-developed photodetector was unable to detect light that had a power density less than 1.58 × 10^−4^ W/cm^2^. The most significant reason for the reduction in the photocurrent of the TiS_3_ nanoribbon-based photodetector could be the stability of the material at a higher temperature. As shown in the TGA graph of Figure 2b, the weight% of the TiS_3_ nanoribbons decreased to 94%, while the temperature was raised from 443 K to 498 K. This means that the TiS_3_ nanoribbons went under phase change after 443 K. It has been reported that due to the annealing of TiS_3_ nanostructures at higher temperatures, sulfur vacancies start to be created. In addition, TiS_3_ nanostructures start to convert into TiO_2_ [44]. TiO_2_ is known to be a wide-bandgap (~3.2 eV) material, and TiO_2_ does not show any light absorbance in the range of 617 nm [45]. Therefore, the generation of defects in the structure of TiS_3_ and its partial conversion into TiO_2_ makes TiS_3_ less sensitive at elevated temperatures, especially beyond a 498 K temperature. Therefore, this may be considered an important reason for the reduction in the photocurrent in the elevated temperature ranges. In conclusion, we can say that the obtained pattern of the photocurrent, the increment in the photocurrent when the operating temperature is increased from 77 K to 333 K, and then the decrement in the photocurrent with respect to the increment in the operating temperature beyond 333 K, is the combined outcome of various phenomena, such as mobility, dark current, device resistance, charge carrier scattering, non-radiative recombination, and changes in the band structure and material structure with respect to the operating temperature, etc. A kind of trade-off between all of these phenomena was observed. The best suitable combination of all these phenomena was observed at a 333 K operating temperature. The maximum value of the photocurrent was found to be 6.95 × 10^−5^ A at a 333 K of operating temperature using the illumination of a 617 nm wavelength of light with a 9.93 × 10^−4^ W/cm^2^ power density.

**Figure 4:**
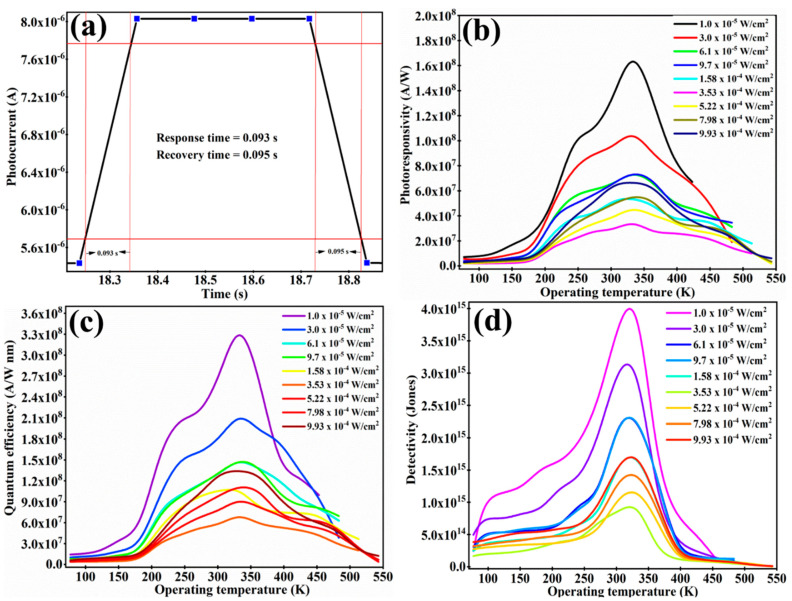
(**a**) Shows the response/recovery plot for the as-developed photodetector at a 273 K operating temperature under the illumination of a 617 nm wavelength with a 1.58 × 10^−4^ W/cm^2^ power density of light. The response/recovery time has been calculated from 10 % to 90% of current (see red line). (**b**–**d**) Represent the plots for the photoresponsivity, quantum efficiency and detectivity as a function of the operating temperature and power density of light.

After analyzing the pattern of the photocurrent, as well as the sensing mechanism behind the production of the photocurrent, the other important parameters for the as-developed photodetector, such as the response/recovery time, photoresponsivity, quantum efficiency, detectivity and device ON/OFF ratio, were observed. Figure 4a shows the room temperature response/recovery time graph for a 1.58 × 10^−4^ W/cm^2^ power density of light. The response and recovery time was calculated from 10% to 90% of the photocurrent. The values of the response and recovery time were found to be 93 ms and 95 ms, respectively. The value of the response and recovery time verifies the rapidity of the as-developed photodetector. The values of the response and recovery time were calculated for each power density of light, as well as for the device operating temperature. The response and recovery time for the as-developed photodetector for all sets of experiments were found to be in the range of 93 ms to 200 ms (see Figure S4 (SI)). The value of the device ON/OFF ratio and the ratio between the photocurrent and the dark current, was found to be 32. The value of 32 of the device ON/OFF ratio is quite high and shows the high merit of the as-developed photodetector. 

To obtain a clear picture of the merits of the performance of the as-developed photodetector, it is very important to analyze the photoresponsivity (R_λ_) of the prepared device. Photoresponsivity provides a valuable picture of the input–output gain of a photodetector. It can be calculated using the following relationship between the ∆I (difference between the photocurrent and dark current), P (power density of light) and S (area of the device) [2]:

(4) $$R\lambda_{=}\frac{\Delta I}{P.S}$$

The values of photoresponsivity as a function of the operating temperature and power density of light are plotted in Figure 4b. The maximum value of photoresponsivity was detected for a 333 K operating temperature. The value of photoresponsivity was found to decrease for both sides of the 333 K operating temperature. It started to decrease less and more than the 333 K operating temperature. The pattern of reduction in the photoresponsivity below and above a 333 K operating temperature was found to be different. The slope of reduction in photoresponsivity was found to be slow below a 333 K operating temperature compared to one above 333 K. On the cooling side, the lowest value of photoresponsivity was detected for a 77 K operating temperature. The possible reason for the lower value of photoresponsivity is the non-radiative recombination in the form of an Auger recombination. It is possible that, due to the low mobility of the charge carriers and due to non-radiative recombination, the charge carriers trapped in the energy levels near the band edges start to prevent the absorbance of new photons. In addition, as we explained earlier, as the bandgap energy and structural defects created at the cryogenic temperature increase, the absorbance of new photons decreases. The overall phenomenon creates a material that is less favorable for absorbing light energy and for the generation and separation of charge carriers. Therefore, this could also be a reason for the low photoresponsivity. When the temperature of the device increases, the non-radiative recombination starts to decrease and other factors, such as the increment in the bandgap energy and disturbance in the band structure, start to shift towards its actual situation. That is why photoresponsivity starts to increase slowly. As explained earlier, the behavior of the dark current with respect to the operating temperature remains almost constant for cryogenic reasons; however, above a 333 K operating temperature, it starts to increase sharply. Therefore, the dark current does not play a major role in the cryogenic temperature, but it starts to become a major concern for operating temperatures above 333 K. Due to the sharp increment in the dark current in the positive temperature regions (greater than 333 K), the photoresponsivity decreases sharply. Due to excess charge carrier generation at the elevated temperature range, the energy states near the band edges can be occupied immediately due to the large number of free charge carriers; this situation is less favorable for the absorbance of new photons. This is apart from the fact that the phase shift in the structure of the materials (partial conversion of TiS_3_ to TiO_2_) and scattering of the charge carriers could also be responsible for the reduction in the photoresponsivity at an elevated temperature range. 

Quantum efficiency and detectivity are also used to evaluate the performance of photodetection devices. Quantum efficiency represents the generation of charge carriers with respect to the absorbance of photons, while detectivity demonstrates the ability of a photodetector to detect a weak signal. Quantum efficiency can be defined by the mathematical relationship between the photoresponsivity (R_λ_), wavelength of the incident light (λ), electronic charge (e), Plank’s constant (h) and velocity of light (c) [2]:

(5) $$\mathrm{Quantum}\;\mathrm{efficiency}=\mathrm{hc}\;\frac{R_{\lambda }}{e.\lambda }$$

Meanwhile, detectivity can be expressed by the following mathematical relation between the R_λ_, effective area of the device (A_d_), dark current (I_d_) and e [2]:

(6) $$\mathrm{Detectivity}=\frac{R_{\lambda }.A_{d}^{1/2}}{{\left(2e.I_{d}\right)}^{1/2}}$$

Figure 4c,d shows the plots for quantum efficiency and detectivity as a function of the operating temperature and power density of light, respectively. The pattern for quantum efficiency and detectivity with respect to the operating temperature was found to be the same as that for photoresponsivity. The maximum value of quantum efficiency and detectivity was found at a 333 K operating temperature; it starts to decrease as the value of the operating temperature is either increased or decreased compared to 333 K. The reason behind this pattern of quantum efficiency and detectivity can be considered the same as that which is explained for photocurrent and photoresponsivity. In the case of detectivity, the dark current plays a major role; that is why the detectivity of the device decreases sharply at elevated temperatures compared to the photoresponsivity and quantum efficiency. The various effects of the power density of light on photoresponsivity, quantum efficiency and detectivity were explained in our previous article [28]. Here, our focus is only on the various effects of the operating temperature on the performance of the device. The highest value of photoresponsivity, quantum efficiency and detectivity was observed for a 333 K operating temperature, at 1.624 × 10^8^ A/W, 3.3 × 10^8^ A/W·nm and 4.328 × 10^15^ Jones, respectively. The values of photoresponsivity, quantum efficiency and detectivity are very high, which verifies the high performance of the as-developed photodetector. All the measurements were conducted for a 617 nm laser light. However, TiS_3_ nanoribbons show a very strong absorbance of light from UV–Vis–IR region (see Figure 2a). Therefore, we believe that TiS_3_ nanoribbons can be used for the development of broadband (UV–Vis–NIR) photodetectors with a wide range of operating temperatures. The as-developed photodetector is able to detect a broad range in the power density of light. It can detect the signal with a very small power density (1.1 × 10^−5^ W/cm^2^) of light. Table 1 shows a performance comparison between the photodetector in the present work and other reported devices. The majority of photodetection parameters used for the TiS_3_ nanoribbon-based photodetector are equal to or better than the reported work.

## 4. Conclusions

An ultra-sensitive photodetector for a 617 nm wavelength of light with a high photocurrent (6.95 × 10^−5^ A), photoresponsivity (1.624 × 10^8^ A/W), quantum efficiency (3.3 × 10^8^ A/W·nm) and detectivity (4.328 × 10^15^ Jones) has been developed for a wide range of operating temperatures (77 K–543 K). A detailed study of the electronic and optoelectronic properties of TiS_3_ nanoribbons, and especially of their temperature-dependent photodetection properties, has been conducted. Their temperature-dependent light-sensing mechanism has been established using the proper justification and evidence. The as-developed a photodetector is capable of detecting very low intensities of light in the broad region of the solar spectrum. The as-developed photodetector shows a very quick response (response/recovery time ~93 ms) and device ON/OFF ratio (~32) within a wide operating temperature range.

Before the fabrication of the solid-state device, TiS_3_ nanoribbons were fabricated using the CVT technique and investigated for their morphological, structural, electronic and optoelectronic properties via SEM, TEM, Raman spectroscopy, XRD, a UV–Vis–NIR spectrophotometer and TGA. The solid-state device for photodetection application was developed using the DEP technique. To the best of our knowledge, it is the first report on a TiS_3_ nanoribbon-based photodetector that can be used in a wide operating temperature range. We believe that the as-developed photodetector that can be used within a wide operating temperature range and its study will be helpful for the development of modern optoelectronic devices for aeronautical and cryogenic applications. 

## Figures and Tables

**Table 1:** Performance comparison between the photodetector in the present work and other reported devices.

S. No.	Material	Operating Temperature (K)	Wavelength (nm)	Power Density of Light (W/cm^2^)	Photocurrent (A)	Photoresponsivity (A/W)	Response/Recovery Time (s)	Detectivity (Jones)	Ref.
1.	MoS_2_	Room temperature	561	238	~10^−5^	880	4/9	-	[46]
2.	PbS colloidal quantum dot	Room temperature	975	80 pW	-	1000	in ms	1.8 × 10^13^	[47]
3.	CH_3_NH_3_PbI_3_	373	400–800	0.00659	~10^−5^	-	0.04/0.05	-	[48]
4.	Metal-halide perovskite nanowires	Room temperature	530	8.7 × 10^−8^	~10^−4^	1.5 × 10^4^	27.6 × 10^−6^/24.5 × 10^−6^	7 × 10^15^	[49]
5.	CdTe	Room temperature	500	5 × 10^−12^	~10^−4^	4 × 10^9^	10 × 10^−3^/10	5 × 10^17^	[50]
6.	Graphene	Room temperature	850	-	-	7	-	-	[51]
7.	TiS_2_ nanosheets	Room temperature	455	0.0792	1.34 × 10^−5^	1.174 × 10^4^	0.3/0.18	3.039 × 10^11^	[52]
8.	TiS_2_ nanosheets	93–423	617	7.92 × 10^−4^	4.52 × 10^−8^	8.9 × 10^4^	0.099/0.099	1.93 × 10^14^	[2]
9.	ZnS–MoS_2_	Room temperature	780	1.91 × 10^−2^	1.5 × 10^−6^	4.5 9 × 10^–6^	31/30	-	[53]
10.	MAPbI_3_	Room temperature	-	0.145	~10^−5^	37.14	9.1 × 10^−5^/5.63 × 10^−4^	2.06 × 10^13^	[54]
11.	TiS_3_ nanoribbon	77 K–543 K	617	1 × 10^−5^–9.93 × 10^−4^	6.95 × 10^−5^	1.624 × 10^8^	0.093/0.093	4.328 × 10^15^	Present work

## Data Availability

No new data were created or analyzed in this study. Data sharing is not applicable to this article.

## Supplementary Materials

The following supporting information can be downloaded at: https://www.mdpi.com/article/10.3390/s23104948/s1, Figure S1: It shows the real-time photocurrent vs. power density of light plots for different operating temperature (77 K–303 K). The measurements have been conducted with 5 V bias voltage with 617 nm laser light. The values of power density of light in W/cm^2^ have been taken as following: P1 = 1.0 × 10^−5^; P2 = 3.0 × 10^−5^; P3 = 6.1 × 10^−5^; P4 = 9.7 × 10^−5^; P5 = 1.58 × 10^−4^; P6 = 3.53 × 10^−4^; P7 = 5.22 × 10^−4^; P8 = 7.98 × 10^−4^; P9 = 9.93 × 10^−4^. Figure S2: Figure shows the real-time photocurrent vs. power density of light plots for different operating temperature (333 K–543 K). The measurements have been conducted with 5 V bias voltage with 617 nm laser light. The values of power density of light in W/cm^2^ have been taken as following: P1 = 1.0 × 10^−5^; P2 = 3.0 × 10^−5^; P3 = 6.1 × 10^−5^; P4 = 9.7 × 10^−5^; P5 = 1.58 × 10^−4^; P6 = 3.53 × 10^−4^; P7 = 5.22 × 10^−4^; P8 = 7.98 × 10^−4^; P9 = 9.93 × 10^−4^. Figure S3: Figure shows the I-V characteristics for the dark conditions for different values of operating temperature. Figure S4: Plot for the response and recovery time.
