# Supplementary material for: TiS3 Nanoribbons: A Novel Material for Ultra-Sensitive Photodetection across Extreme Temperature Ranges
Source: Sensors (Basel). 2023 May 21;23(10):4948. doi: 10.3390/s23104948 (PMC10221207; doi:10.3390/s23104948)
Supplement: Supplementary file 1 [file sensors-23-04948-s001.zip › sensors-2371776-supplementary.pdf]

sensors

Supplementary Material

TiS_3-nanoribbon: A Novel Material for Ultra-Sensitive Photo-detection Across Extreme Temperature Ranges

Mohammad Talib[1], Nishant Tripathi[2*], Samrah Manzoor[1], Prachi Sharma[2,4], Vladimir Pavelyev[2,5], Valentyn S. Volkov[3], Aleksey V. Arsenin[3,6], Sergey M. Novikov[3], Prabhash Mishra[1,2,*]

[1]*Centre for Nanoscience and Nanotechnology, Jamia Millia Islamia (A Central University), New Delhi 110025, India.*

[2]*Samara National Research University, 34, Moskovskoye Shosse, Samara, 443086, Russia.*

[3]*Center for Photonics & 2D Materials, Moscow Institute of Physics and Technology (MIPT), Dolgoprudny 141700, Russia.*

[4]*School of Electronics Engineering (SENSE), Vellore Institute of Technology (VIT), Vellore, Tamil Nadu 632014, India.*

[5]*IPSI RAS – Branch of the FSRC "Crystallography and Photonics" RAS, 443001, Samara, Russia, Molodogvardeyskaya 151.*

[6]*Laboratory of Advanced Functional Materials, Yerevan State University, Yerevan, 0025, Armenia*

[*]Corresponding Authors email: nishant.tripathi.11@gmail.com (Nishant Tripathi), pmishra@jmi.ac.in (Prabhash Mishra)

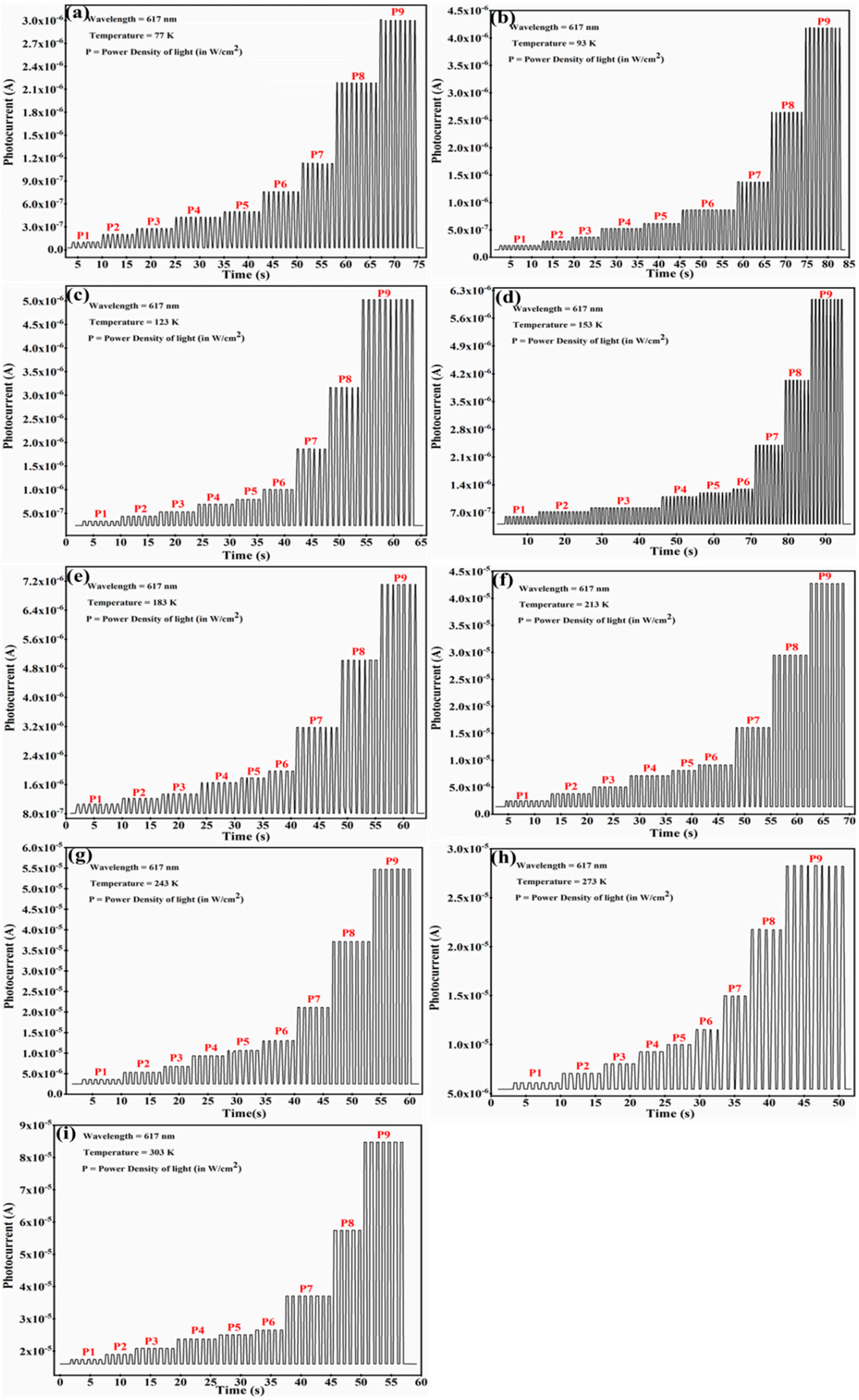

Figure S1: It shows the real-time photocurrent vs. power density of light plots for different operating temperature (77 K-303 K). The measurements have been conducted with 5 V bias voltage with 617 nm laser light. The values of power density of light in

W/cm^2 have been taken as following: P1 = 1.0 X 10-5; P2 = 3.0 X 10-5; P3 = 6.1 X 10-5; P4 = 9.7 X 10-5; P5 = 1.58 X 10-4; P6 = 3.53 X 10-4; P7 = 5.22 X 10-4; P8 = 7.98 X 10-4; P9 = 9.93 X 10-4.

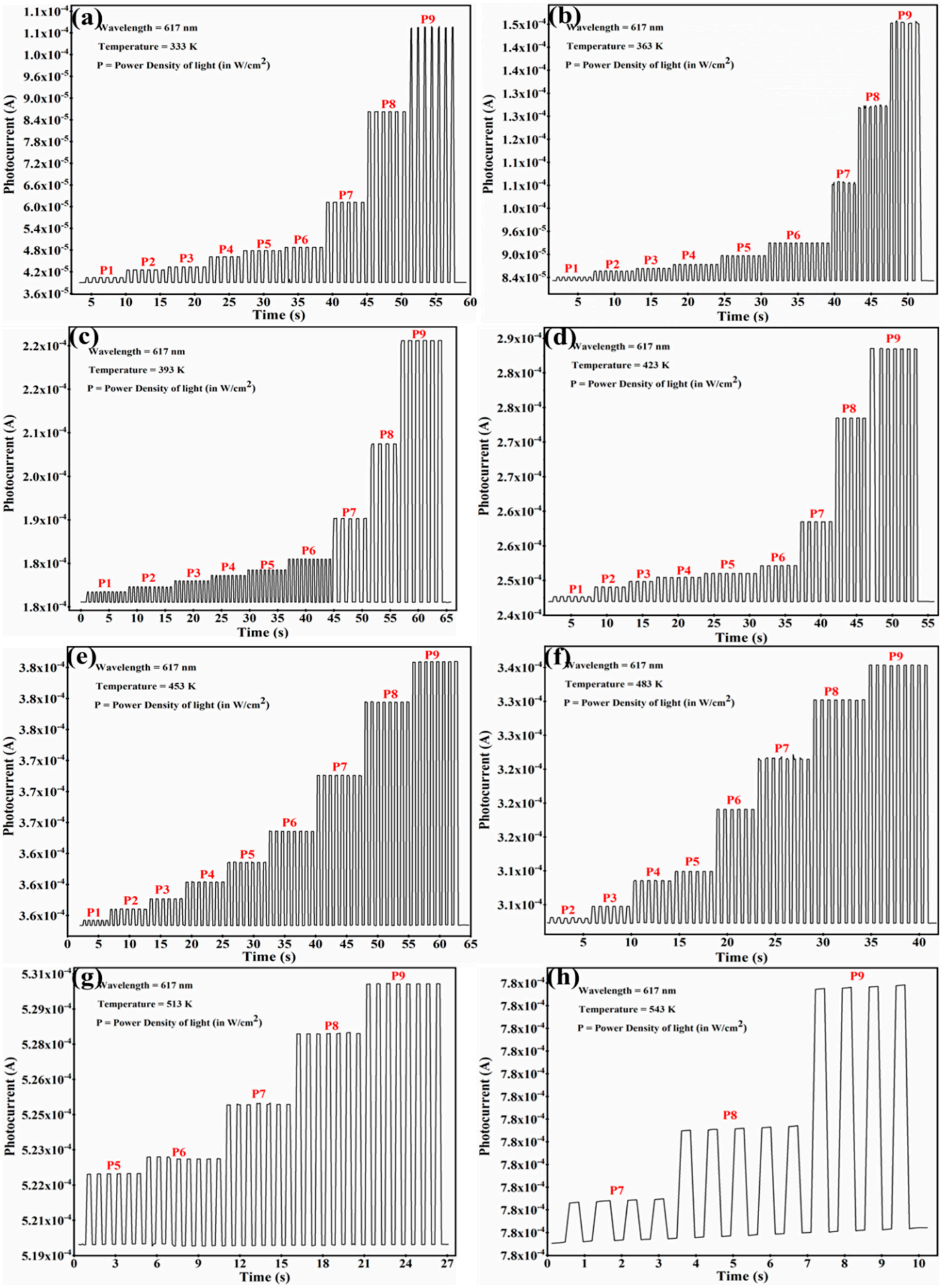

Figure S2: Figure shows the real-time photocurrent vs. power density of light plots for different operating temperature (333 K-543 K). The measurements have been conducted with 5 V bias voltage with 617 nm laser light. The values of power density of light in W/cm^2 have been taken as following: P1 = 1.0 X 10-5; P2 = 3.0 X 10-5; P3 = 6.1 X 10-5; P4 = 9.7 X 10-5; P5 = 1.58 X 10-4; P6 = 3.53 X 10-4; P7 = 5.22 X 10-4; P8 = 7.98 X 10-4; P9 = 9.93 X 10-4.

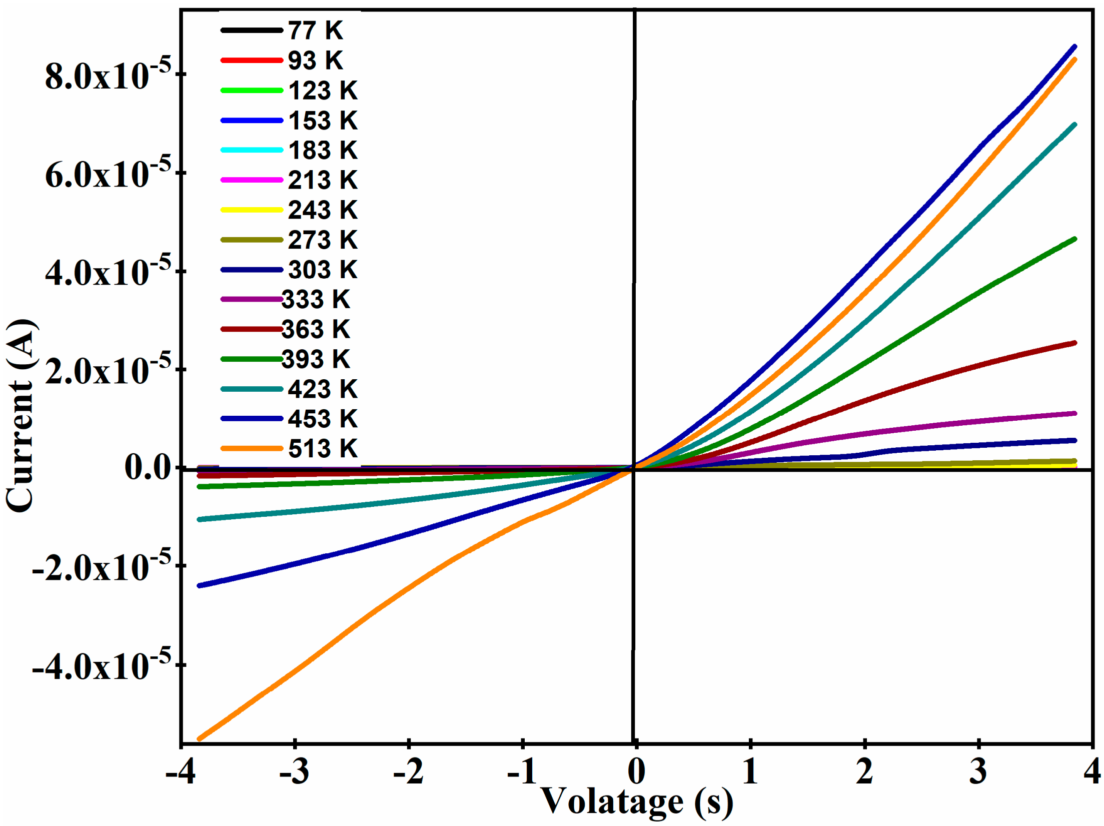

Figure S3: Figure shows the I-V characteristics for the dark conditions for different values of operating temperature.

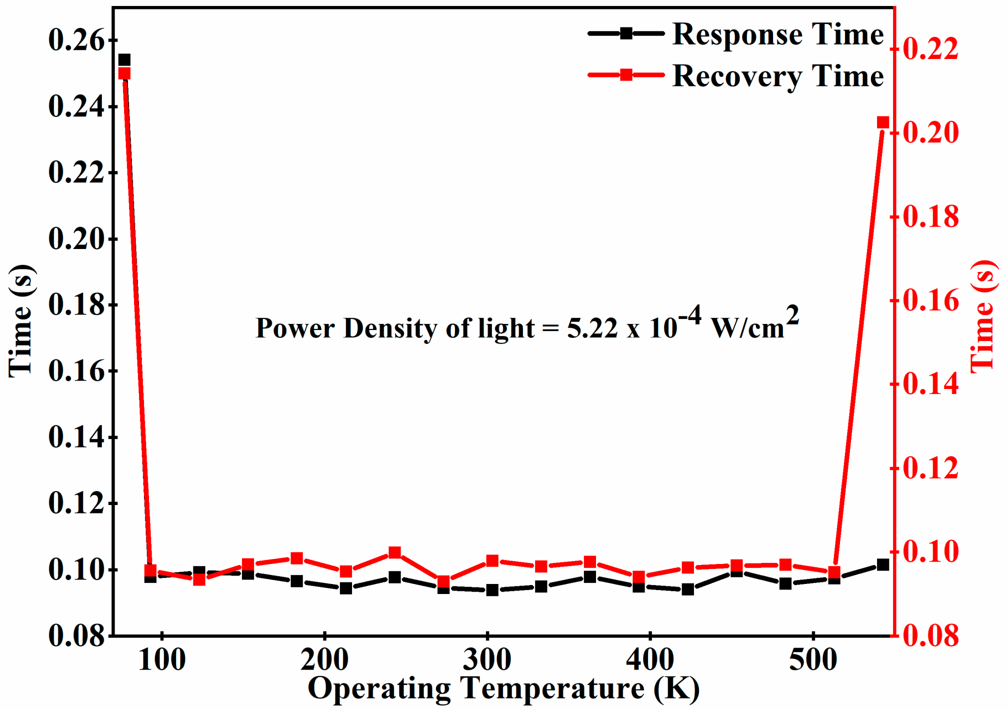

Figure S4: Plot for the response and recovery time.
